# Social inequalities in all-cause mortality among adults with multimorbidity: a 10-year prospective study of 0.5 million Chinese adults

**DOI:** 10.1093/inthealth/ihac052

**Published:** 2022-08-03

**Authors:** Siyu Zou, Zhicheng Wang, Kun Tang

**Affiliations:** Vanke School of Public Health, Tsinghua University, 30 Shuangqing Road, Haidian District, Beijing 100084, China; School of Public Health, Peking University Health Science Center, 38 Xueyuan Road, Haidian District, Beijing 100191, China; Vanke School of Public Health, Tsinghua University, 30 Shuangqing Road, Haidian District, Beijing 100084, China; Vanke School of Public Health, Tsinghua University, 30 Shuangqing Road, Haidian District, Beijing 100084, China

**Keywords:** all-cause mortality, inequalities, multimorbidity, prospective cohort study, socio-economic status

## Abstract

**Background:**

Chinese individuals face an increase in multimorbidity, but little is known about the mortality gradients of multimorbid people in different socio-economic groups. This study measures relative and absolute socio-economic inequality in mortality among multimorbid Chinese.

**Methods:**

For this study, the prospective China Kadoorie Biobank (CKB) enrolled 512 712 participants ages 30–79 y from 10 areas of China between 25 June 2004 and 15 July 2008. All-cause mortality was accessed with a mean follow-up period of 10 y (to 31 December 2016). Associations between multimorbidity and mortality were assessed using Cox proportional hazards models, with the relative index of inequality (RII) and slope index of inequality (SII) in mortality calculated to measure disparities.

**Results:**

Mortality risk was highest for those who had not attended formal school and with four or more long-term conditions (LTCs) (hazard ratio 3.11 [95% confidence interval {CI} 2.75 to 3.51]). Relative educational inequality was lower in participants with four or more LTCs (RII 1.92 [95% CI 1.60 to 2.30]), especially in rural areas. Absolute disparities were greater in adults with more LTCs (SII 0.18 [95% CI 0.14 to 0.21] for rural participants with three LTCs).

**Conclusions:**

Whereas the relative inequality in all-cause mortality was lower among multimorbid people, absolute inequality was greater among multimorbid men, especially in rural areas.

## Introduction

Non-communicable diseases (NCDs) are the world's leading causes of death, accounting for 72% (40 million) of the world's 55 million deaths in 2016.^1^ The World Health Organization's (WHO) 2014 global status report on NCDs in 194 countries states that 75% of NCD deaths occur in low- and middle-income countries (LMICs) such as China, the world's largest middle-income country.^2^ The Global Burden of Disease Study shows that China's most common NCDs, including stroke, cancer and chronic obstructive pulmonary disease, are the country's top causes of death (7.9 million), accounting for 87% of total deaths in 2013.^3^ The burden of NCDs was predicted to be high and NCD complications impose an enormous burden on China's public health services, with multimorbid persons having a mortality rate approximately four times higher than those without.^4^ Multimorbidity, usually defined as the coexistence of two or more NCDs within an individual,^5^ is now the norm rather than the exception for people with chronic disease in China.^6^ In 2015, a nationally representative survey of middle-aged and older Chinese people reported the overall prevalence of physical multimorbidity was 61·9%.^7^

Studies of high-income countries identify the risk factors for multimorbidity as increasing age, a high number of previous diseases, female gender and low socio-economic status (SES).^8,9^ The public health burden of multimorbidity is unevenly distributed across socio-economic strata^10^: among people with NCDs, SES may influence major health determinants such as access to care, quality of care and health behaviours.^11^ Correspondingly, SES may have profound impacts on morbidity and mortality.^12^ People defined as low SES by their income, wealth, education or living area are more likely to die young than people with a high SES.^13^ A large, population-based study of multimorbidity inequalities has shown that socially disadvantaged men become multimorbid 2 y earlier and, after becoming multimorbid, survive 1 y less than their advantaged counterparts.^14^ Other studies show education level to be a risk factor for developing long-term disease.^15^ China has an ageing population, meaning that the proportion of people with coexisting medical problems is increasing rapidly.^16^ Healthcare expenditures increase almost exponentially with the number of chronic disorders suffered, meaning that increased multimorbidity generates financial pressures.^16,17^

After an epidemiologic transition^18^ characterized by a decline in mortality rates combined with an ageing population^1^ in high-income countries, NCD risk factors are shown to be typically associated with economic disadvantage and low education levels. However, this trend is often different in cases of LMICs,^19^ which have various gradations of the double burden of infectious diseases and NCDs, with the balance shifting inexorably towards NCDs.^19,20^ Still, evidence from LMICs remains limited.

In China, few studies have focused on SES-related disparities among multimorbid individuals, while previous studies report inconsistent associations between SES and multimorbidity prevalence in China.^21^ For example, some studies report increased multimorbidity prevalence with high education levels,^7^ while others find significant inequalities and higher concentrations of multimorbidity among lower-SES individuals in China.^22,23^ Most evidence on multimorbidity comes from studies with inconsistent design, small sample size or specific populations in different settings. Consequently, improved research on socio-economic inequalities in multimorbidity and its effect on all-cause mortality is urgently needed.

Valid estimations on associations between multimorbidity and mortality require a comparison of multimorbid people with similar individuals who do not suffer from NCDs. Few population cohorts have sufficient power, detail and longevity to enable such comparisons, making it difficult to determine the impact of such disparities at the population level and their importance for public health.^24^ To fully monitor these health disparities, both relative and absolute measures are required. The objective of this study was to quantify the relative and absolute SES disparities as a cause of mortality within multimorbid Chinese populations, comparing the magnitude of these disparities with non-multimorbid individuals.

## Methods

### Study design and participants

Data from the China Kadoorie Biobank (CKB) study, a large-scale prospective cohort of >0.5 million participants ages 30–79 y, was used for this study. Detailed information on CKB's study design, survey methods and population has been reported previously.^25,26^ The baseline survey occurred between 25 June 2004 and 15 July 2008 in 10 geographical areas (five urban and five rural, defined according to governmental administrative structure) of China. These areas were selected from China's nationally representative Disease Surveillance Points (DSPs) as maximizing regional and social diversity, levels of SES, exposure to certain risk factors, population stability, quality of death and disease registries and long-term local commitment to the project. Official local residential records were used to identify individuals eligible for this study (adults ages 30–79 y). All participants completed an interviewer-administered electronic questionnaire providing detailed information on their demographic and socio-economic characteristics, medical history and lifestyle factors. Physical measurements were also recorded and a blood sample collected for long-term storage. Overall, 512 715 individuals (including a small number of individuals outside the target age range) agreed to participate in the study. One participant was excluded from the study due to a very high number (≥16) of coexisting diseases, while two participants were excluded due to missing baseline body height or weight values. The final analyses included 512 712 individuals comprised of 210 203 (41.0%) men and 302 509 (59.0%) women (see Table 1).

**Table 1.** Characteristics of 512 172 participants with and without multimorbidity, according to education level

	Adults without multimorbidity (n=431 352)				Adults with multimorbidity (n=81 360)			
		
Variables	No formal school	Primary school	Middle or high school	College and above	No formal school	Primary school	Middle or high school	College and above
		
		
All participants	75 350 (17.47)	136 908 (31.74)	194 321 (45.05)	24 773 (5.74)	19 822 (24.36)	28 274 (34.75)	28 060 (34.49)	5204 (6.40)
Sex								
Female	60 840 (80.74)	79 765 (58.26)	103 571 (53.30)	11 545 (46.60)	15 670 (79.05)	15 304 (54.13)	13 920 (49.61)	1894 (36.40)
Male	14 510 (19.26)	57 143 (41.74)	90 750 (46.70)	13 228 (53.40)	4152 (20.95)	12 970 (45.87)	14 140 (50.39)	3310 (63.60)
Age (years)								
30–39	4315 (5.73)	20 276 (14.81)	42 356 (21.80)	7650 (30.88)	184 (0.93)	868 (3.07)	1642 (5.85)	321 (6.17)
40–49	13 812 (18.33)	30 181 (22.04)	87 996 (45.28)	8605 (34.74)	1457 (7.35)	2639 (9.33)	7210 (25.69)	854 (16.41)
50–59	29 085 (38.6)	52 478 (38.33)	44 742 (23.02)	4542 (18.33)	5890 (29.71)	10 284 (36.37)	9296 (33.13)	1293 (24.85)
60–69	18 302 (24.29)	26 915 (19.66)	16 180 (8.33)	2915 (11.77)	7378 (37.22)	10 694 (37.82)	7504 (26.74)	1868 (35.90)
70–79	9836 (13.05)	7058 (5.16)	3047 (1.57)	1061 (4.28)	4913 (24.79)	3789 (13.40)	2408 (8.58)	868 (16.68)
Annual income (CNY)								
<10 000	33 599 (44.59)	46 745 (34.14)	39 425 (20.29)	558 (2.25)	9038 (45.60)	10 272 (36.33)	4973 (17.72)	124 (2.38)
10 000–19 999	16 230 (21.54)	42 387 (30.96)	64 400 (33.14)	3231 (13.04)	4343 (21.91)	8578 (30.34)	9007 (32.10)	780 (14.99)
20 000–34 999	14 556 (19.32)	29 721 (21.71)	55 099 (28.35)	8189 (33.06)	3629 (18.31)	5715 (20.21)	8178 (29.14)	1614 (31.01)
≥35 000	10 965 (14.55)	18 055 (13.19)	35 397 (18.22)	12 795 (51.65)	2812 (14.19)	3709 (13.12)	5902 (21.03)	2686 (51.61)
Employment status								
Employed	51 633 (68.52)	98 657 (72.06)	131 966 (67.91)	18 304 (73.89)	10 007 (50.48)	14 587 (51.59)	11 352 (40.46)	1993 (38.30)
Unemployed	18 071 (23.98)	23 320 (17.03)	29 396 (15.13)	1275 (5.15)	6796 (34.29)	6387 (22.59)	3878 (13.82)	152 (2.92)
Retired	5646 (7.49)	14 931 (10.91)	32 959 (16.96)	5194 (20.97)	3019 (15.23)	7300 (25.82)	12 830 (45.72)	3059 (58.78)
Region								
Rural	55 538 (73.71)	102 094 (74.57)	86 858 (44.70)	2717 (10.97)	12 751 (64.33)	17 746 (62.76)	8434 (30.06)	392 (7.53)
Urban	19 812 (26.29)	34 814 (25.43)	107 463 (55.30)	22 056 (89.03)	7071 (35.67)	10 528 (37.24)	19 626 (69.94)	4812 (92.47)
BMI								
<18.5	4597 (6.10)	6889 (5.03)	6289 (3.24)	684 (2.76)	1368 (6.90)	1644 (5.81)	785 (2.80)	104 (2.00)
18.5–24.9	41 220 (54.7)	76 340 (55.76)	101 637 (52.30)	12 285 (49.59)	9190 (46.36)	12 661 (44.78)	10 665 (38.01)	1878 (36.09)
25–29.9	22 574 (29.96)	41 544 (30.34)	66 667 (34.31)	9306 (37.57)	6427 (32.42)	9771 (34.56)	11 525 (41.07)	2323 (44.64)
≥30	6959 (9.24)	12 135 (8.86)	19 728 (10.15)	2498 (10.08)	2837 (14.31)	4198 (14.85)	5085 (18.12)	899 (17.28)
Smoking status								
Non-smoker	59 418 (78.86)	81 664 (59.65)	112 937 (58.12)	15 102 (60.96)	14 724 (74.28)	15 472 (54.72)	15 350 (54.70)	2818 (54.15)
Ex-smoker	2509 (3.33)	7649 (5.59)	9666 (4.97)	1464 (5.91)	1567 (7.91)	3544 (12.53)	3371 (12.01)	791 (15.20)
Current smoker	13 423(17.81)	47 595 (34.76)	71 718 (36.91)	8207 (33.13)	3531 (17.81)	9258 (32.74)	9339 (33.28)	1595 (30.65)
Alcohol consumption								
Non-drinker	53 294 (70.73)	66 984 (48.93)	68 170 (35.08)	5077 (20.49)	14 414 (72.72)	14 605 (51.66)	11 120 (39.63)	1438 (27.63)
Ex-drinker	911 (1.21)	2874 (2.10)	1865 (0.96)	154 (0.62)	702 (3.54)	1672 (5.91)	932 (3.32)	145 (2.79)
Current drinker	21 145(28.06)	67 050(48.97)	124 286 (63.96)	19 542 (78.88)	4706 (23.74)	11 997 (42.43)	16 008 (57.05)	3621 (69.58)
Physical activity								
Activity	38 543 (51.15)	59 264 (43.29)	86 145 (44.33)	6454 (26.05)	6371 (32.14)	7550 (26.70)	7036 (25.07)	622 (11.95)
Inactivity	36 807 (48.85)	77 644 (56.71)	108 176 (55.67)	18 319 (73.95)	13 451 (67.86)	20 724 (73.30)	21 024 (74.93)	4582 (88.05)

### Follow-up for mortality

Information on all-cause mortality was collected periodically from baseline until 31 December 2016. Data were collected through linkages via a unique national identification number using China's DSP system. This system was supplemented by local social health insurance databases and annual validation of survival using local residential and administrative records.

### Multimorbidity coding

Earlier studies defined multimorbidity as the concurrence of two or more LTCs in the same individual. Consistent with previous literature^27^ and considering the common LTCs reported by the National Health Services Survey,^28^ 16 LTCs were selected for this study: asthma, emphysema/bronchitis, chronic obstructive pulmonary disease (COPD), hypertension, diabetes, coronary heart disease, stroke, peptic ulcer, cirrhosis/chronic hepatitis, gallstone/gallbladder disease, kidney disease, rheumatic heart disease, rheumatoid arthritis, psychiatric disorders, neurasthenia and cancer.^10^ These 16 conditions were identified by participants’ self-reported diagnoses and supplemented by medical examinations. Participants were asked at baseline, ‘Has a doctor ever told you that you have the following diseases?’ Those who reported ‘yes’ were defined as having self-reported the kind of LTC previously. For example, dementia would be considered a psychiatric disorder while a history of cancer indicated all kinds of cancers, including that of the lung, oesophagus, stomach, liver, intestine, breast, prostate, cervix and elsewhere. Additionally, a 10-mL blood sample was collected from study participants at baseline.^10^

### Assessment of SES

SES was measured by education and income: highest level of an individual's school achievement (categorised as no formal school, primary school, middle or high school and college and above) and annual household income (<10 000, 10 000–19 999, 20 000–34 999 and ≥35 000 Chinese yuan [CNY]).

### Assessment of covariates

Relevant covariates included demographic characteristics (age, sex, residence), medical insurance, health behaviours (smoking status, alcohol consumption, physical activity) and anthropometric indicators (body mass index [BMI]) (see Supplementary Methods).

### Statistical analysis

Descriptive statistics are presented in Table 1 for anthropometric and lifestyle characteristics, subdivided by multimorbidity status and education level. Education and income disparities for all-cause mortality in people with multimorbidity were measured using Cox regression models, controlling for confounding variables^12^ such as age, sex, region, education level, household income, occupation, smoking and alcohol status, physical activity levels and BMI, where appropriate. Three indicators were used to estimate absolute and relative socio-economic inequalities in mortality. First, hazard ratios (HRs) of death associated with different numbers of LTCs were computed, using people with no LTC as a reference. While HRs are easy to interpret, comparisons of HRs across various population groups are complicated by different distributions of education levels across subgroups; indeed, the advantages conferred by factors such as holding a high school degree likely differs across age, sex or region. Use of the relative index of inequality (RII) and slope index of inequality (SII) as measures of socio-economic inequalities overcomes this problem by providing continuous inequality measures that simultaneously account for the size and relative position of education and income groups, using specific measures of individuals’ relative socio-economic position (i.e. mean proportion of the overall population with an education level higher than the participant). Since absolute and relative measures can result in different conclusions on the size of and changes in inequalities, examining both measures is important for providing a complete picture of inequalities.^29^ In contrast with common measures comparing only extreme groups (e.g. the richest and poorest wealth quintiles) such as rate difference and ratio, the RII and SII measure inequality across the entire distribution of socio-economic positions.

The RII and SII were estimated using a generalized linear model (GLM), with the initial choice of link functions based on knowledge of the dataset and conditional distribution of the response variable.^30^ The response variable, all-cause mortality, has a binomial distribution. As in previous literature, GLM (binomial regression) was used with a logarithmic link function to calculate RIIs and an identity link function to calculate SIIs.^31,32^ Models comprised a sex-specific relative education ridit score and income ridit score regressed on current all-cause mortality. The RII may be interpreted as a prevalence ratio and the SII as the prevalence difference between the hypothetical bottom vs top of the education hierarchy. An RII >1 or an SII >0 indicates a higher prevalence in lower vs higher educational groups, an RII <1 or SII <0 indicates a higher prevalence in higher vs lower educational groups and an RII=1 or SII=0 indicates no inequality.

To determine the proportional hazards assumption for the Cox model, graphs and tests based on Schoenfeld residuals were applied, where no violation was identified. The goodness-of-fit of the GLM was assessed by using residual plots. Associations were regarded as statistically significant if p-values were ≤0.05. All analyses were performed using Stata 16 MP (StataCorp, College Station, TX, USA).

## Results

### Characteristics of the study population

The study identified 512 712 adults ages 30–79 y at the time of the CKB interview, with a median follow-up time of 10 y (9.93±1.82 y). At baseline, nearly 34.5% of the study population had one LTC, 11.6% had two LTCs, 3.2% had three LTCs and 1.1% had four or more LTCs. A total of 81 360 (15.6%) participants reported having multimorbidity, the majority of whom were of lower educational and annual income levels. Of this number, 19 822 (24.4%) participants had no formal school and 28 274 (34.8%) had a primary school degree (Table 1). Most participants were in the <10 000 CNY income group (30.0%) and 10 000–19 999 CNY income group (27.9%).

### Relative and absolute disparities in all-cause mortality

All-cause mortality among participants with no LTC in the lowest education level was more than five times greater (RII 5.27 [95% CI 4.84 to 5.73]) than among participants in the highest education level (Table 2). Among participants with four or more LTCs in the lowest education level, all-cause mortality was twice as great (RII 1.92 [95% CI 1.60 to 2.30]) as among participants in the highest education level. The magnitude of both relative education and household income disparities in all-cause mortality was significantly higher in adults with fewer LTCs, especially among women (Table 2, Supplementary Table 1).

**Table 2.** Relative and absolute educational disparities in all-cause mortality among adults with and without multimorbidity

		All-cause mortality		
Variables	Count of LTCs	SII (95% CI)^a^	RII (95% CI)^b^	HR (95% CI)^c^
Total	No LTC (n=254 733)	0.04 (0.04 to 0.04)	5.27 (4.84 to 5.73)	1.00
	1 LTC (n=176 619)	0.09 (0.09 to 0.10)	3.84 (3.60 to 4.08)	2.50 (2.43 to 2.57)
	2 LTC (n=59 225)	0.14 (0.13 to 0.15)	2.88 (2.67 to 3.10)	4.74 (4.59 to 4.89)
	3 LTC (n=16 604)	0.15 (0.13 to 0.17)	2.41 (2.14 to 2.71)	6.61 (6.34 to 6.89)
	≥4 LTC (n=5531)	0.14 (0.10 to 0.18)	1.92 (1.60 to 2.30)	8.02 (7.55 to 8.53)
Male				
	No LTC (n=99 932)	0.07 (0.07 to 0.07)	7.32 (6.53 to 8.19)	1.00
	1 LTC (n=75 699)	0.14 (0.13 to 0.15)	5.40 (4.97 to 5.87)	2.63 (2.43 to 2.83)
	2 LTC (n=25 326)	0.21 (0.20 to 0.23)	3.84 (3.47 to 4.24)	4.83 (4.45 to 5.24)
	3 LTC (n=7059)	0.21 (0.18 to 0.24)	2.60 (2.23 to 3.03)	6.89 (6.23 to 7.62)
	≥4 LTC (n=2187)	0.26 (0.20 to 0.33)	2.46 (1.95 to 3.10)	8.35 (7.33 to 9.50)
Female				
	No LTC (n=154 801)	0.03 (0.03 to 0.03)	7.16 (6.24 to 8.21)	1.00
	1 LTC (n=100 920)	0.08 (0.08 to 0.09)	6.42 (5.78 to 7.12)	2.16 (2.02 to 2.30)
	2 LTC (n=33 899)	0.14 (0.13 to 0.15)	4.53 (4.00 to 5.14)	4.19 (3.90 to 4.49)
	3 LTC (n=9545)	0.18 (0.16 to 0.21)	4.28 (3.51 to 5.22)	6.19 (5.66 to 6.77)
	≥4 LTC (n=3344)	0.15 (0.11 to 0.19)	2.74 (2.04 to 3.67)	8.92 (7.96 to 9.98)
Rural				
	No LTC (n=99 932)	0.05 (0.04 to 0.05)	4.10 (4.54 to 3.70)	1.00
	1 LTC (n=75 699)	0.10 (0.10 to 0.11)	3.20 (3.45 to 2.96)	1.63 (1.58 to 1.69)
	2 LTC (n=25 326)	0.14 (0.13 to 0.16)	2.30 (2.53 to 2.09)	2.43 (2.34 to 2.54)
	3 LTC (n=7059)	0.18 (0.14 to 0.21)	2.10 (2.46 to 1.79)	2.99 (2.82 to 3.16)
	≥ 4 LTC (n=2187)	0.13 (0.06 to 0.21)	1.57 (2.06 to 1.20)	3.59 (3.27 to 3.93)
Urban				
	No LTC (n=154 801)	0.03 (0.03 to 0.03)	4.89 (5.67 to 4.23)	1.00
	1 LTC (n=100 920)	0.05 (0.05 to 0.06)	3.05 (3.41 to 2.74)	1.54 (1.46 to 1.62)
	2 LTC (n=33 899)	0.07 (0.06 to 0.08)	2.15 (2.43 to 1.90)	2.23 (2.11 to 2.36)
	3 LTC (n=9545)	0.06 (0.04 to 0.09)	1.60 (1.91 to 1.34)	2.76 (2.57 to 2.96)
	≥4 LTC (n=3344)	0.06 (0.02 to 0.11)	1.42 (1.81 to 1.11)	3.25 (2.97 to 3.55)

a SII: absolute difference in predicted mortality rates between the lowest (ridit score of 0) and the highest (ridit score of 1) values of the distribution of socio-economic characteristic (by education).

b RII: ratio of mortality rates of individuals with the lowest and highest education level in the population. GLM with negative binomial distribution and log link function were used.

c HR: hazard ratio of all-cause mortality (reference category: individuals with no LTC).

In absolute terms, the difference in estimated risk of mortality between adults in the lowest vs those in the highest position, as measured by the SII, was highest among males with four or more LTCs (SII 0.26 [95% CI 0.20 to 0.33] on the education scale and 0.26 [95% CI 0.19 to 0.33] on the household income scale) (Table 2, Supplementary Table 1). These absolute educational disparities in all-cause mortality were greater in adults living in rural areas (e.g. SII 0.18 [95% CI 0.14 to 0.21] for participants with three LTCs) (Table 2).

### All-cause mortality, stratified by education level

The highest crude mortality rate was observed in participants with no formal school and four or more LTCs (28.98 per 1000 person-years), while the lowest was found among those with college degrees and no LTCs (1.36 per 1000 person-years) (Table 3). When stratified by education level, the gap in all-cause mortality between no LTCs and increased number of LTCs persisted and was increased from 5.58 (95% CI 4.96 to 6.29; unadjusted HRs in people with four or more LTCs) for people with no formal schooling to 11.2 (95% CI 8.64 to 14.52) for people with college degrees and above (Table 3). HRs decreased greatly after additional adjustment for age, sex and region but remained statistically significant for any comparison between different LTC groups. Among participants with no formal school the mortality rate was 211% higher for people with four or more LTCs as compared with those with no LTC (HR 3.11 [95% CI 2.75 to 3.51]) (Table 3).

**Table 3.** The relationship between SES and multimorbidity in all-cause mortality according to education level

SES categories	LTCs, n	Participants, n	Deaths, n	Person-years	Mortality rate^a^	Unadjusted, HR (95% CI)	Model 1, HR (95% CI)	Model 2, HR (95% CI)	Model 3, HR (95% CI)
No formal school	0	36 291	1921	368 110	5.22	1.00	1.00	1.00	1.00
	1	39 059	4247	384 829	11.04	2.12 (2.01 to 2.23)	1.55 (1.47 to 1.64)	1.53 (1.44 to 1.61)	1.56(1.48 to 1.65)
	2	14 425	2617	135 997	19.24	3.68 (3.47 to 3.91)	2.34 (2.20 to 2.48)	2.26 (2.13 to 2.41)	2.27 (2.14 to 2.42)
	3	4179	990	38 112	25.98	4.98 (4.62 to 5.38)	2.96 (2.73 to 3.20)	2.85 (2.64 to 3.09)	2.84 (2.63 to 3.08)
	≥4	1218	319	11 008	28.9	5.58 (4.96 to 6.29)	3.33 (2.96 to 3.76)	3.16 (2.80 to 3.56)	3.11 (2.75 to 3.51)
Primary school	0	76 914	3074	775 724	3.96	1.00	1.00	1.00	1.00
	1	59 994	5196	588 994	8.82	2.22 (2.13 to 2.32)	1.60 (1.53 to 1.68)	1.58 (1.51 to 1.66)	1.61 (1.54 to 1.69)
	2	20 824	3150	196 267	16.05	4.03 (3.84 to 4.24)	2.46 (2.34 to 2.59)	2.39 (2.27 to 2.52)	2.37 (2.25 to 2.49)
	3	5737	1141	52 219	21.85	5.48 (5.12 to 5.86)	3.04 (2.83 to 3.26)	2.95 (2.75 to 3.16)	2.88 (2.68 to 3.09)
	≥4	1713	419	15 290	27.40	6.9 (6.23 to 7.64)	3.66 (3.30 to 4.06)	3.51 (3.16 to 3.89)	3.35 (3.01 to 3.72)
Middle or high school	0	125 558	2380	1 271 072	1.87	1.00	1.00	1.00	1.00
	1	68 763	3113	686 458	4.53	2.42 (2.30 to 2.56)	1.67 (1.58 to 1.77)	1.66 (1.58 to 1.76)	1.68 (1.59 to 1.78)
	2	20 498	1857	199 127	9.33	4.97 (4.68 to 5.28)	2.63 (2.47 to 2.81)	2.58 (2.42 to 2.76)	2.57 (2.41 to 2.75)
	3	5501	716	52 139	13.73	7.32 (6.73 to 7.96)	3.21 (2.94 to 3.51)	3.17 (2.90 to 3.46)	3.10 (2.83 to 3.38)
	≥4	2061	377	18 998	19.84	10.55 (9.47 to 11.77)	4.04 (3.60 to 4.52)	4.02 (3.58 to 4.50)	3.92 (3.49 to 4.39)
College and above	0	15 970	218	159 894	1.36	1.00	1.00	1.00	1.00
	1	8803	322	87 695	3.67	2.71 (2.28 to 3.22)	1.44 (1.20 to 1.71)	1.43 (1.20 to 1.71)	1.45 (1.21 to 1.74)
	2	3478	278	33 903	8.20	6.06 (5.07 to 7.23)	2.24 (1.85 to 2.70)	2.21 (1.83 to 2.66)	2.20 (1.82 to 2.66)
	3	1187	153	11 369	13.46	9.99 (8.13 to 12.29)	2.88 (2.31 to 3.58)	2.80 (2.25 to 3.48)	2.76 (2.21 to 3.44)
	≥4	539	77	5080	15.16	11.2 (8.64 to 14.52)	2.90 (2.21 to 3.81)	2.86 (2.18 to 3.75)	2.78 (2.11 to 3.65)
Total	0	254 733	7593	2 574 799	2.95	1.00	1.00	1.00	1.00
	1	176 619	12 878	1 747 977	7.37	2.50 (2.43 to 2.57)	1.61 (1.56 to 1.66)	1.59 (1.54 to 1.63)	1.62 (1.57 to 1.67)
	2	59 225	7902	565 293	13.98	4.74 (4.59 to 4.89)	2.45 (2.37 to 2.54)	2.39 (2.32 to 2.47)	2.39 (2.31 to 2.47)
	3	16 604	3000	153 839	19.50	6.61 (6.34 to 6.89)	3.02 (2.89 to 3.16)	2.97 (2.84 to 3.11)	2.93 (2.80 to 3.06)
	≥4	5531	1192	50 377	23.66	8.02 (7.55 to 8.53)	3.47 (3.26 to 3.70)	3.50 (3.28 to 3.72)	3.40 (3.19 to 3.63)

Model 1: adjusted for age, sex and region.

Model 2: model 1 plus adjustment for household income and employment status.

Model 3: model 2 plus adjustment for BMI, smoking, alcohol consumption and physical activity.

### All-cause mortality in people with multimorbidity, stratified by sex and region

**Figure 1.**
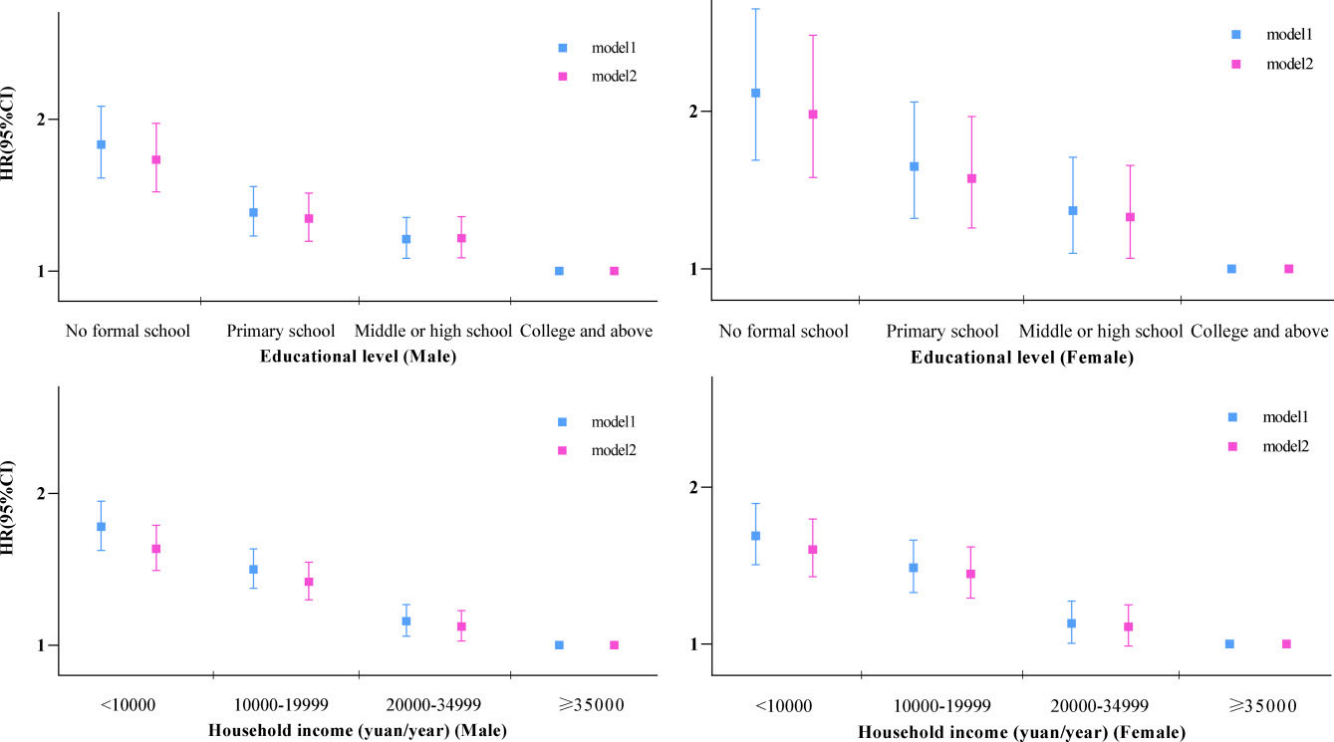
Adjusted HRs and 95% CIs for all-cause mortality in people with multimorbidity, associated with **(a, b)** education level and **(c, d)** household income in **(a, c)** men and **(b, d)** women. For education level, model 1 was adjusted for age at baseline, study region, household income and employment status; model 2 added BMI, smoking, alcohol consumption and physical activity. For household income, model 1 was adjusted for age at baseline, study region, education level and employment status; model 2 added BMI, smoking, alcohol consumption and physical activity.

**Figure 2.**
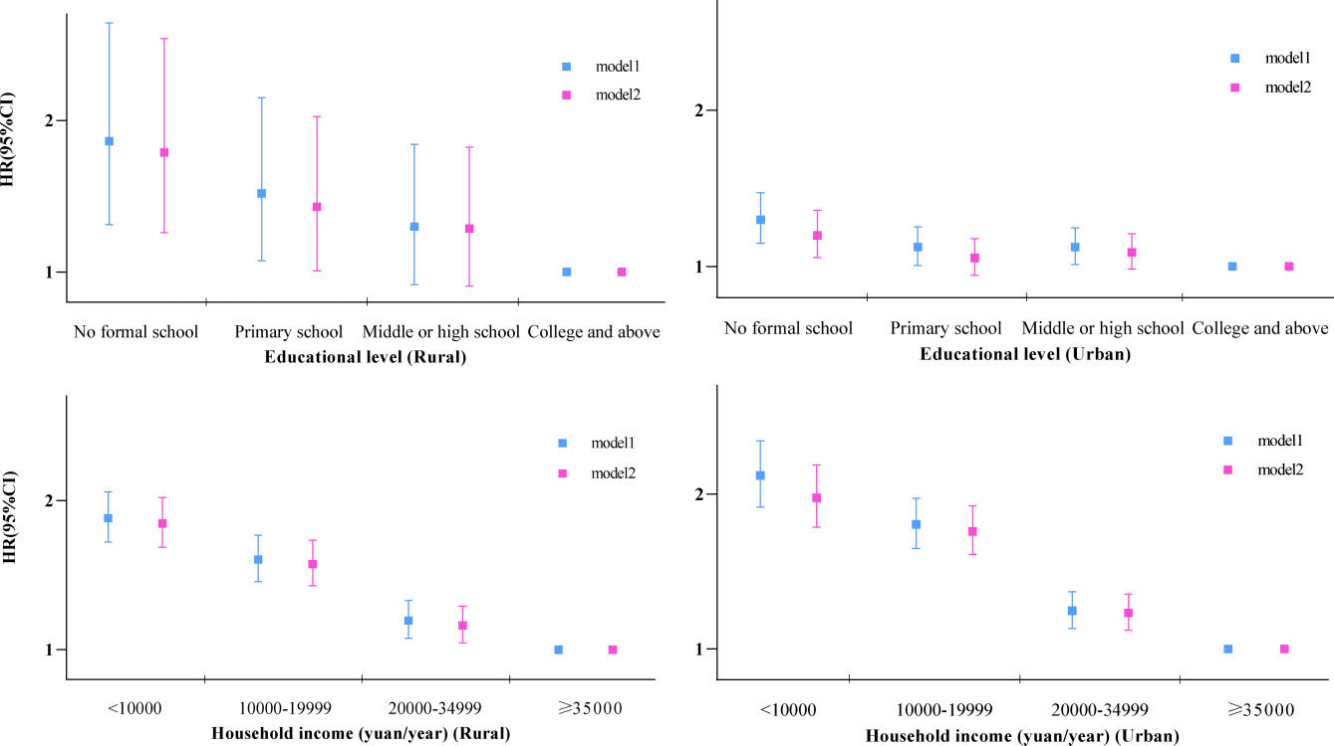
Adjusted HRs and 95% CIs for mortality in people with multimorbidity associated with **(a, b)** education level and **(c, d)** household income in **(a, c)** rural and **(b, d)** urban areas. For education level, model 1 was adjusted for age at baseline, study region, household income and employment status; model 2 added BMI, smoking, alcohol consumption and physical activity. For household income, model 1 was adjusted for age at baseline, study region, education level and employment status; model 2 added BMI, smoking, alcohol consumption and physical activity.

When stratified by sex and region, education level was negatively associated with all-cause mortality in people with multimorbidity (Figures 1 and 2). Model 1 showed a more significantly inverse association in women and rural areas. Adjustment for health behaviours and health indicators led to a slight decrease in people with different education levels. Among individuals from the same SES levels (i.e. the same education level or household income), a greater number of LTCs indicated a higher risk of death (Supplementary Figures 1 and 2).

## Discussion

This large nationwide prospective study of around 0.5 million Chinese adults showed that differences in SES have a substantial association with mortality risk in multimorbid participants, regardless of the number of LTCs, sex or region. In absolute terms, multimorbid adults with lower education or household income levels had a higher mortality burden, especially among males and those living in rural areas. The magnitude of relative disparities in all-cause mortality was significantly higher in adults with fewer LTCs, especially in women.

Evidence for socio-economic disparities in mortality among persons with multimorbidity from LMICs is rare. To our knowledge, this study is the first nationwide prospective study to explore the effect of SES on the association of multimorbidity and all-cause mortality in China. Based on a previous study that found people with a lower SES have a higher prevalence of multimorbidity,^10^ this research reports more deaths with an increased number of LTCs and lower SES, which is consistent with other studies from high-income countries.^13,33,34^

This study also found that absolute disparities in mortality among multimorbid individuals are strong, specifically for people with four or more LTCs. Relative disparities in mortality decreased with an increasing number of LTCs, a finding similar to that of a Danish study.^15^ One possible explanation for this finding is that when individuals had a relatively small number of LTCs, high SES provided those people with more available resources for early detection and treatment.^35^ Social differences arose during the relatively small number of LTCs stage, mainly because knowledge on how to prevent multimorbidity favours high-status groups who are quicker to acquire new information and change their health behaviours.^36^ A greater number of LTCs increased the difficulty of disease management and effective treatment, while the difficulty of multimorbidity management levels off disparities in healthcare and health behaviours across various educational groups.^37^ A systematic review of 26 studies found a positive gradient between the number of LTCs and mortality, concluding that these relationships are complex and suffer effect modification due to socio-economic factors.^38^ Moreover, most studies conducted in China are narrowly concerned with multimorbidity prevalence in different SES groups.^17,22^ For example, a nationally representative survey found that physical multimorbidity was more common in the country's most deprived rather than its most affluent regions.^7^ Prospective studies are warranted on the impact of SES on all-cause mortality in people with multimorbidity. The present study shows that mortality risk differs substantially according to education level, a finding consistent with previous studies in New Zealand, Europe and the UK.^6,12,39^

When stratified by sex and region, the education and household income levels of participants with multimorbidity showed negative associations with the risk of all-cause mortality. Consistent with most previous literature, absolute education inequality was generally higher and relative education inequality lower for men than women.^40^ The reason for this finding is not yet clear, as a range of factors may be at play.^41^ Lower absolute education inequality in women was observed in this study and may be attributable to both biological and modifiable factors such as lower risk-taking, smoking and alcohol consumption.^42^ Higher relative education inequality levels in women may be at least partly attributable to lower SES levels, resulting in a lack of sufficient knowledge on preventing NCDs.^43^ Additionally, different sexes may have a different disease spectrum, and previous studies have found that women have a more complex network of disease associations than men.^44,45^

This study found education disparity to be a prime explanation for health inequalities among urban and rural adults. In both absolute and relative terms, findings indicated that disparities in all-cause mortality are more apparent among participants living in rural areas, a difference already analysed in detail in the Global Burden of Disease study.^46^ Chinese individuals in urban areas generally have better access to higher levels of education and increased awareness of healthy behaviours.^47^ Moreover, allocation of health resources is uneven between urban and rural areas, with China's healthcare service provision inefficient among China's rural areas.^48^ The urbanisation of China's population may have several varied health effects, but further research is needed on the risks and benefits of urban vs rural lifestyles.^49^

The contrasting results obtained for relative or absolute disparity measures stem from the fact that the burden of all-cause mortality is dramatically higher in multimorbid adults as compared with adults without multimorbidity. This finding highlights the relevance of using both relative and absolute inequality measures for adequately assessing health disparities and suggests that educational health disparities among multimorbid adults have a major public health impact.

### Strengths and limitations

This study's strengths, including a national cohort large enough to afford multiple stratified multivariate analyses and a long-term follow-up period that is nearly 100% complete, lend weight to its conclusions. A key strength of this study is that it is the first nationwide study describing contemporary social inequalities in its associations between multimorbidity and all-cause mortality in China. There are two important advantages in using the RII and SII.^50^ First, these incorporate changes to the size of education level and income groups by regressing rates on the midpoint of each group's rank on a scale of 0 to 1 (i.e. ridit score). The RII and SII capture the impact of changing education levels and income distribution, as well as change mortality disparities by actual unit. Second, the RII and SII are regression-based measures that utilise mortality rate estimates for all six education levels and income groups rather than just compare extreme groups.

This study has several limitations. First, reliance on self-reporting LTCs may underestimate their prevalence, particularly among older people and those from lower socioeconomic and educational backgrounds who are more likely to underreport these factors.^51^ Consequently, educational health disparities measured among adults with self-reported multimorbidity may be less marked than those occurring among the whole population of persons with multimorbidity. Second, different SES metrics may be intertwined and thus influence risk factors for health or disease at different points in a person's life.^52^ While low SES is often assumed to have causal effects on health, the direction of causality is more likely to be reversed. Despite this, several empirical studies using quasi-experimental designs have identified a causal effect of SES on health and mortality,^53^ and results remain stable after further adjustments for health behaviours and indicators in our study. Additionally, previous studies have shown that there is no single best indicator of SES suitable for all study aims and applicable to all times in all settings.^54^ Compared with only one indicator as a marker of SES, both education level and household income were used in this study. Third, we showed a higher mortality risk in people with higher LTCs. The mechanisms behind this result may be related to different multimorbid patterns^55^ but are unclear, and future studies are needed to assess whether people with different SES have different multimorbid patterns.

## Conclusions

By showing that the risk of all-cause mortality differs according to education level and household income, both in relative and absolute terms, the results of the present study provide strong evidence for the existence of socio-economic inequalities in mortality among Chinese individuals with different numbers of LTCs. While the effects of relative educational disparities on mortality are less marked in adults with multimorbidity than in those without, their absolute impact is greater. Considering the major burden of multimorbidity in China, especially among the most deprived categories of males and rural residents, this study's findings suggest that reducing social health inequalities among multimorbid individuals will likely have major public health implications. Future research should determine pathways underlying these socio-economic inequalities, including patient factors (e.g. sex, region, health behaviours or material conditions), with an eye towards developing elimination strategies, as well as characteristics of patient providers, communities and the healthcare system.

## Supplementary Material

ihac052_Supplemental_Files

## Data Availability

Details of the CKB data used in this study are available from the CKB upon reasonable request (http://www.ckbiobank.org/site/Data+Access).
