# Supplementary material for: Social inequalities in all-cause mortality among adults with multimorbidity: a 10-year prospective study of 0.5 million Chinese adults
Source: Int Health. 2022 Aug 3;15(2):123–33. doi: 10.1093/inthealth/ihac052 (PMC9977254; doi:10.1093/inthealth/ihac052)
Supplement: ihac052_Supplemental_Files [file ihac052_supplemental_files.zip › Supplement figure.docx]

## Supplementary Figure 1. Adjusted HRs and 95% CIs for all-cause mortality in people with different LTCs, stratified by socioeconomic status and sex.

Educational level (a, b) and household income (c, d) in men (a, c) and women (b, d), plotted on a logarithmic scale.

Abbreviations: BMI = body mass index; LTC = long-term conditions.

For educational level, model was adjusted for age at baseline, study region, household income, employment status, BMI, smoking, alcohol consumption, and physical activity.

For household income, model was adjusted for age at baseline, study region, educational level, employment status, BMI, smoking, alcohol consumption, and physical activity.

## Supplementary Figure 2. Adjusted HRs and 95% CIs for all-cause mortality in people with different LTCs, stratified by SES and region.

Educational level (a, b) and household income (c, d) in rural (a, c) and urban (b, d), plotted on a logarithmic scale.

Abbreviations: BMI = body mass index; LTC = long-term conditions.

For educational level, model was adjusted for age at baseline, sex, household income, employment status, BMI, smoking, alcohol consumption, and physical activity.

For household income, model was adjusted for age at baseline, sex, educational level, employment status, BMI, smoking, alcohol consumption, and physical activity.
