# Supplementary material for: Social inequalities in all-cause mortality among adults with multimorbidity: a 10-year prospective study of 0.5 million Chinese adults
Source: Int Health. 2022 Aug 3;15(2):123–33. doi: 10.1093/inthealth/ihac052 (PMC9977254; doi:10.1093/inthealth/ihac052)
Supplement: ihac052_Supplemental_Files [file ihac052_supplemental_files.zip › Supplement information.docx]

## Social inequalities in all-cause mortality among adults with multimorbidity in China: a 10-year follow-up study of 0.5 million Chinese adults

## Section S1. Supplementary Methods: Baseline questionnaires

### Health behaviors (smoking history, alcohol consumption, physical activity)

**Smoking history**

Individuals were classified by smoking status as non-smoker, ex-smoker, or current smokers. Non-smokers were defined as individuals who reported not smoking at baseline and had smoked <100 cigarettes (or equivalent) in their lifetime. Ex-smokers were defined as individuals who had smoked ≥100 cigarettes (or equivalent) but had quit smoking by choice for ≥6 months before baseline. Current smokers were defined as individuals who reported having smoked ≥1 cigarettes (or equivalent) daily for at least 6 months at baseline, or individuals who did not meet the criteria for never smokers and who had not stopped smoking completely for ≥6 months before baseline.

**Alcohol consumption**

Drinking categories were non-drinker, ex- drinker, and current drinkers. Non-drinkers were defined as individuals who had never or almost never consumed alcohol in the past year and had not drunk weekly in the past. Ex-drinkers were individuals who had drunk weekly in the past but had never or almost never consumed alcohol in the past year. Current drinkers were defined as occasional or seasonal, reduced-intake, and weekly drinkers.

**Physical activity**

Physical activity was estimated by summing the metabolic equivalent task (MET) hours per day (MET-h/day) spent on usual type and duration of occupational and nonoccupational activities (transportation, housework, and leisure-time exercise) during the past year. Details of the measurements have been described elsewhere[1]. We grouped physical activity into active and inactive (<21.1 MET-h/day [median of total physical activity in all participants]).

### Health indicator (body mass index)

Body mass index (BMI) at baseline was defined as the measured weight (kg) divided by the square of the measured height (m). BMI was used as a categorical variable with 4 categories (<18.5, 18.5 to <24.0, 24.0 to <28.0, and ≥28.0 kg/m^2^).
