# Supplementary material for: Social inequalities in all-cause mortality among adults with multimorbidity: a 10-year prospective study of 0.5 million Chinese adults
Source: Int Health. 2022 Aug 3;15(2):123–33. doi: 10.1093/inthealth/ihac052 (PMC9977254; doi:10.1093/inthealth/ihac052)
Supplement: ihac052_Supplemental_Files [file ihac052_supplemental_files.zip › Supplement table.docx]

## Supplementary Table 1. Relative and absolute income disparities in all-cause mortality among adults with and without multimorbidity.

|  | **Count of LTCs** | **All-cause mortality** | | | | | | | |
| --- | --- | --- | --- | --- | --- | --- | --- | --- | --- |
|  |  | **SII (95% CI)^1^** | |  | **RII (95% CI)^2^** | |  | **HR(95%CI)^3^** | |
| **Total** | No LTC N = 254733 | -0.03 | -0.04,-0.03 |  | 3.88 | 4.22,3.58 |  | 1.00 | |
|  | 1 LTC N = 176619 | -0.10 | -0.10,-0.09 |  | 4.72 | 5.03,4.44 |  | 1.62 | 1.57,1.67 |
|  | 2 LTC N = 59225 | -0.17 | -0.18,-0.16 |  | 4.14 | 4.46,3.84 |  | 2.39 | 2.31,2.47 |
|  | 3 LTC N = 16604 | -0.20 | -0.21,-0.18 |  | 3.18 | 3.58,2.83 |  | 2.93 | 2.80,3.06 |
|  | ≥ 4 LTC N = 5531 | -0.19 | -0.23,-0.15 |  | 2.47 | 2.96,2.06 |  | 3.40 | 3.19,3.63 |
|  |  |  |  |  |  |  |  |  |  |
| **Male** |  |  |  |  |  |  |  |  |  |
|  | No LTC N = 99932 | -0.05 | -0.06,-0.05 |  | 4.00 | 4.45,3.60 |  | 1.00 | |
|  | 1 LTC N = 75699 | -0.14 | -0.15,-0.13 |  | 5.37 | 5.82,4.95 |  | 1.63 | 1.56,1.71 |
|  | 2 LTC N = 25326 | -0.23 | -0.25,-0.22 |  | 4.20 | 4.63,3.81 |  | 2.50 | 2.37,2.63 |
|  | 3 LTC N = 7059 | -0.24 | -0.27,-0.20 |  | 2.80 | 3.25,2.41 |  | 3.12 | 2.91,3.34 |
|  | ≥ 4 LTC N = 2187 | -0.26 | -0.33,-0.19 |  | 2.36 | 2.96,1.88 |  | 3.45 | 3.13,3.80 |
|  |  |  |  |  |  |  |  |  |  |
| **Female** |  |  |  |  |  |  |  |  |  |
|  | No LTC N = 154801 | -0.02 | -0.03,-0.02 |  | 4.34 | 4.94,3.80 |  | 1.00 | |
|  | 1 LTC N = 100920 | -0.07 | -0.08,-0.07 |  | 4.84 | 5.34,4.39 |  | 1.60 | 1.54,1.67 |
|  | 2 LTC N = 33899 | -0.14 | -0.15,-0.13 |  | 4.77 | 5.37,4.24 |  | 2.29 | 2.19,2.39 |
|  | 3 LTC N = 9545 | -0.18 | -0.20,-0.16 |  | 4.40 | 5.31,3.64 |  | 2.77 | 2.61,2.94 |
|  | ≥ 4 LTC N = 3344 | -0.17 | -0.21,-0.13 |  | 3.23 | 4.30,2.42 |  | 3.32 | 3.05 ,3.61 |
|  |  |  |  |  |  |  |  |  |  |
| **Rural** |  |  |  |  |  |  |  |  |  |
|  | No LTC N = 144974 | -0.03 | -0.04,-0.03 |  | 3.12 | 3.45,2.83 |  | 1.00 | |
|  | 1 LTC N = 102233 | -0.11 | -0.11,-0.10 |  | 4.08 | 4.40,3.79 |  | 1.63 | 1.58,1.69 |
|  | 2 LTC N = 30242 | -0.17 | -0.19,-0.16 |  | 3.06 | 3.36,2.79 |  | 2.43 | 2.33,2.54 |
|  | 3 LTC N = 7282 | -0.18 | -0.21,-0.14 |  | 2.28 | 2.65,1.96 |  | 2.99 | 2.82 ,3.16 |
|  | ≥ 4 LTC N = 1799 | -0.17 | -0.24,-0.09 |  | 1.82 | 2.36,1.40 |  | 3.58 | 3.27,3.93 |
|  |  |  |  |  |  |  |  |  |  |
| **Urban** |  |  |  |  |  |  |  |  |  |
|  | No LTC N = 109759 | -0.03 | -0.04,-0.03 |  | 3.88 | 4.22,3.58 |  | 1.00 | |
|  | 1 LTC N = 74386 | -0.10 | -0.10,-0.09 |  | 4.72 | 5.03,4.44 |  | 1.54 | 1.46,1.62 |
|  | 2 LTC N = 28983 | -0.17 | -0.18,-0.16 |  | 4.14 | 4.46,3.84 |  | 2.23 | 2.11,2.36 |
|  | 3 LTC N = 9322 | -0.20 | -0.21,-0.18 |  | 3.18 | 3.58,2.83 |  | 2.76 | 2.57,2.96 |
|  | ≥ 4 LTC N = 3732 | -0.19 | -0.23,-0.15 |  | 2.47 | 2.96,2.06 |  | 3.25 | 2.97,3.55 |

Abbreviations: BMI = body mass index; LTC = long-term conditions.

^1^SII: Slope Index of Inequality. Absolute difference in predicted mortality rates between the lowest (ridit score of 0) and the highest (ridit score of 1) values of the distribution of socioeconomic characteristic (by household income).

^2^RII: Relative Index of Inequality. Ratio of mortality rates of individuals with the lowest and highest educational level in the population

GLM with Negative Binomial distribution and log link function were used.

^3^HR: Hazard Ratio of all-cause mortality (Reference category: individuals with no LTC)

## Supplementary Table 2. The relationship between socioeconomic status and multimorbidity in all-cause mortality, according to household income.

| **SES categories** | **Count of LTCs** | **No. of Participants** | **No. of Deaths** | **Person-Years** | **Mortality Rate^a^** |  | **Unadjusted** |  | **Model 1** |  | **Model 2** |  | **Model 3** |  |
| --- | --- | --- | --- | --- | --- | --- | --- | --- | --- | --- | --- | --- | --- | --- |
|  |  |  |  |  |  |  | **HR (95%CI)** |  | **HR (95%CI)** |  | **HR (95%CI)** |  | **HR (95%CI)** |  |
|  |  |  |  |  |  |  |  |  |  |  |  |  |  |  |
| <10000 | No LTC | 68844 | 3116 | 705912 | 4.41 |  | 1.00 |  | 1.00 |  | 1.00 |  | 1.00 |  |
|  | 1 LTC | 51483 | 5900 | 507884 | 11.62 |  | 2.63(2.52-2.75) |  | 1.74(1.66-1.82) |  | 1.70(1.62-1.77) |  | 1.72(1.64-1.80) |  |
|  | 2 LTC | 18113 | 3577 | 169819 | 21.06 |  | 4.75(4.53-4.99) |  | 2.65(2.52-2.78) |  | 2.53(2.41-2.66) |  | 2.49(2.36-2.62) |  |
|  | 3 LTC | 4824 | 1216 | 43578 | 27.90 |  | 6.30(5.89-6.73) |  | 3.23(3.02-3.46) |  | 3.07(2.87-3.29) |  | 2.99(2.79-3.21) |  |
|  | ≥4 LTC | 1470 | 412 | 13261 | 31.07 |  | 7.06(6.37-7.83) |  | 3.60(3.24-3.99) |  | 3.40(3.06-3.78) |  | 3.27(2.94-3.63) |  |
|  |  |  |  |  |  |  |  |  |  |  |  |  |  |  |
| 10000-19999 | No LTC | 76255 | 2281 | 775085 | 2.94 |  | 1.00 |  | 1.00 |  | 1.00 |  | 1.00 |  |
|  | 1 LTC | 49993 | 3552 | 498111 | 7.13 |  | 2.43(2.30-2.56) |  | 1.56(1.48-1.65) |  | 1.54(1.46-1.63) |  | 1.58(1.49-1.67) |  |
|  | 2 LTC | 16468 | 2288 | 157596 | 14.52 |  | 4.93(4.65-5.22) |  | 2.52(2.37-2.68) |  | 2.48(2.33-2.63) |  | 2.49(2.34-2.65) |  |
|  | 3 LTC | 4647 | 895 | 42916 | 20.85 |  | 7.07(6.55-7.64) |  | 3.08(2.84-3.34) |  | 3.04(2.80-3.29) |  | 3.01(2.78-3.27) |  |
|  | ≥4 LTC | 1593 | 379 | 14349 | 26.41 |  | 8.98(8.05-10.01) |  | 3.62(3.23-4.05) |  | 3.64(3.25-4.07) |  | 3.55(3.17-3.98) |  |
|  |  |  |  |  |  |  |  |  |  |  |  |  |  |  |
| 20000-34999 | No LTC | 64289 | 1363 | 645555 | 2.11 |  | 1.00 |  | 1.00 |  | 1.00 |  | 1.00 |  |
|  | 1 LTC | 43276 | 2195 | 429496 | 5.11 |  | 2.43(2.27-2.60) |  | 1.57(1.46-1.68) |  | 1.55(1.45-1.67) |  | 1.60(1.49-1.71) |  |
|  | 2 LTC | 13853 | 1279 | 134059 | 9.54 |  | 4.52(4.19-4.88) |  | 2.28(2.11-2.47) |  | 2.24(2.07-2.43) |  | 2.27(2.09-2.46) |  |
|  | 3 LTC | 3916 | 531 | 37055 | 14.33 |  | 6.81(6.16-7.53) |  | 2.85(2.57-3.17) |  | 2.81(2.53-3.12) |  | 2.81(2.53-3.13) |  |
|  | ≥4 LTC | 1367 | 241 | 12610 | 19.11 |  | 9.04(7.89-10.37) |  | 3.46(3.00-3.99) |  | 3.49(3.03-4.02) |  | 3.48(3.01-4.02) |  |
|  |  |  |  |  |  |  |  |  |  |  |  |  |  |  |
| ≥35000 | No LTC | 45345 | 833 | 448247 | 1.86 |  | 1.00 |  | 1.00 |  | 1.00 |  | 1.00 |  |
|  | 1 LTC | 31867 | 1231 | 312485 | 3.94 |  | 2.12(1.95-2.32) |  | 1.34(1.22-1.47) |  | 1.34(1.22-1.46) |  | 1.36(1.24-1.49) |  |
|  | 2 LTC | 10791 | 758 | 103819 | 7.30 |  | 3.93(3.56-4.34) |  | 1.91(1.72-2.11) |  | 1.90(1.72-2.11) |  | 1.89(1.70-2.10) |  |
|  | 3 LTC | 3217 | 358 | 30290 | 11.82 |  | 6.38(5.64-7.22) |  | 2.58(2.27-2.94) |  | 2.58(2.27-2.94) |  | 2.54(2.22-2.89) |  |
|  | ≥4 LTC | 1101 | 160 | 10157 | 15.75 |  | 8.49(7.17-10.06) |  | 2.98(2.50-3.55) |  | 3.08(2.59-3.68) |  | 2.99(2.51-3.57) |  |

Model 1: adjusted for age, sex, and region.

Model 2: Model 1 plus adjustment for educational level, and employment status.

Model 3: Model 2 plus adjustment for BMI, smoking, alcohol consumption, and physical activity.

Abbreviations: HR, hazard ratio; CI, confidence interval; Ref, reference; LTCs long-term conditions; BMI = body mass index
